# Identification and genomic characterization of a novel HIV-1 unique recombinant form (CRF01_AE/CRF07_BC) in Zhejiang Province, China

**DOI:** 10.1007/s11262-022-01945-1

**Published:** 2022-10-27

**Authors:** Qin Fan, Jing Liu, Chengliang Chai, Shuying Zhu, Qionglou Fang, Zhihong Guo, Yan Xia, Xiaobei Ding, Jiafeng Zhang

**Affiliations:** 1 Department of HIV/AIDS Control and Prevention, Zhejiang Provincial Center for Disease Control and Prevention, No.3399, Binsheng Road, Hangzhou, 310051 People’s Republic of China; 2 School of Laboratory Medicine and Life Science, Wenzhou Medical University, Wenzhou, 325035 People’s Republic of China; 3 Department of Microbiological Analysis, Jinhua Municipal Center for Disease Control and Prevention, Jinhua, 321002 People’s Republic of China

**Keywords:** HIV-1, Unique recombinant form (URF), Recombinant, Near full-length genome (NFLG)

## Abstract

Mutation and recombination are important mechanisms leading to the frequent evolution and genetic diversity of viruses as HIV-1. In this study, we identified the near full-length genomic characterization of a novel HIV-1 unique recombinant form (URF) strain (Sample ID: ZJ20202195/ZJ/CHN/2020, hereafter referred to as ZJ20202195) isolated during the HIV-1 molecular surveillance in 2020 in Zhejiang Province, China, through different recombination analysis tools and phylogenetic analysis. Our results amply proved that the near full-length genome (NFLG) sequence of ZJ20202195 was a novel HIV-1 unique recombinant form (URF) consisting of CRF01_AE and CRF07_BC subtype, and delimited three recombinant segments, of which the Segment I (HXB2:776–5559 nucleotide (nt)) and Segment III (HXB2:6224–9412 nt) were mainly originated from CRF01_AE cluster g4a strains prevalent in China and Segment II (HXB2:5560–6223 nt) was from CRF07_BC subtype. Overall, our findings provide insight and a scientific basis in the genetic diversity and accurate determination of HIV-1 recombinant strains in China.

For the past 40 years, human immunodeficiency virus type 1 (HIV-1) has been identified as one of the fastest mutating pathogens. The subtype characteristics and genetic diversity are complex, which is directly related to the high mutation rate, recombination rate, and replication rate of HIV-1 [1, 2]. Mutation and recombination are important mechanisms leading to the frequent evolution and genetic diversity of HIV-1 [3]. To date, more than 110 circulating recombinant forms (CRFs) and plenty of unique recombinant forms (URFs) were reported globally (https://www.hiv.lanl.gov/content/sequence/HIV/CRFs/CRFs.html).

China, as one of the countries with a large number of HIV-infected people and complex and diverse HIV-1 subtypes, faces enormous challenges in AIDS prevention and control [4]. The fourth national HIV-1 molecular epidemiological survey conducted in 2016 showed that the types of HIV-1 subtypes in China increased rapidly, became more complex and diversified, and the main HIV-1 subtypes were CRF07_BC, CRF01_AE, accounting for more than 70.0% [5]. Since the 1990s, CRF01_AE and CRF07_BC have undergone continuous evolution in China and have caused a widespread epidemic in various groups (e.g., men who have sex with men (MSM), heterosexual individuals, and people who inject drugs (PWID)) [5–7]. In addition, New CRFs (e.g., CRF55_01B, CRF59_01B, and CRF85_BC) and a large number of URFs consisting of CRF01_AE, B, and (or) C subtypes appear on a small scale among certain groups [8–10]. Therefore, the different characteristics and recombination patterns of HIV-1 subtypes in different regions and populations indicate that China is facing increasingly complex genetic heterogeneity of HIV-1, with the emergence of novel URFs. It should be noted that most of the new HIV-1 subtypes of recombinant were first discovered in China due to intensive nucleic acid sequence analysis [11–13]. Different HIV-1 strains have unique transmission advantages and regional adaptation characteristics, which are also the result of the adaptive selection of HIV-1. New HIV-1 CRFs and URFs have occupied an important position in China and even the global epidemic.

In this study, we analyzed the near full-length genomic characterization of a novel HIV-1 URF isolated in 2020 from a case of HIV-1-positive patient infected by heterosexual contact identified during HIV-1 molecular surveillance in Zhejiang Province. The patient was a 49-year-old man with junior high school education, diagnosed by Jinhua Municipal Center for Disease Control and Prevention in May 2020 and infected through heterosexual transmission and had multiple commercial heterosexual behaviors according to his report. The patient did not undergo antiretroviral therapy before plasma sample collection, and CD4^+^ T-cell count was 69 cells/μl. The sample was named ZJ20202195. This study was reviewed and approved by the Medical Ethics Committee of the National Center for AIDS/STD Control and Prevention (Approval ID: X140617334), and written informed consent was obtained from the study participant. All experiments were performed by following the approved guidelines and regulations.

Viral RNA was extracted from 140-μl plasma sample using the QIAamp® Viral RNA Mini Kit (Qiagen, Hilden, Germany), following the manufacturer’s instructions. Using the extracted RNA as template, RT-PCR and nested PCR were used to amplify the full-length genome sequence of HIV-1 in two segments. The first round of RT-PCR amplification was performed with SuperScript® III One-Step RT-PCR Platinum® Taq HiFi kit (Invitrogen, Life Technologies, MA, USA). In the second round of nested PCR amplification, the KAPA HiFi Hot-Start Ready Mix kit (KAPA Biosystem, MA, USA) was used. The final PCR product consisted of two amplified fragments: fragment 1 (F1) with a length of about 5.5 kb (gag-vpu) and fragment 2 (F2), about 3.6 kb (vif-3’-LTR), a detailed method is described in reference 14 [14]. Target PCR products were sent to the Hangzhou TsingKe Bio-Tech Co. for purification and sequencing, as previously described [6]. Every nucleotide position was sequenced at least once.

The trimming and assembly of sequences were performed using Sequencher® v5.4.6 (Gene Codes, Ann Arbor, MI, USA), and the sequences of the two obtained fragments (F1 and F2) were spliced together to obtain a HIV-1 NFLG sequence of ZJ20202195 with approximately 9.0 kb length. Quality Control analysis of the HIV-1 NFLG sequence of ZJ20202195 was performed using the HIV Sequence Database online analytical tool Quality Control. Based on the reference sequence (HXB2, GenBank ID: K03455), using the online analysis software HIV Sequence Locator (https://www.hiv.lanl.gov/content/sequence/LOCATE/locate.html) to determine the relative position of the obtained HIV-1 NFLG sequence of ZJ20202195. Finally, we realized that the NFLG sequence of ZJ20202195 was 8636 bp (from 776 to 9412 nt), matched to the HXB2 sequence). The NFLG sequence of ZJ20202195 includes all structural genes and regulatory genes (gag-pol-vif-vpr-tat-rev-vpu-env-nef- partial 3’-LTR).

The NFLG sequence of ZJ20202195 was submitted to the Basic Local Alignment Search Tool (BLAST) analysis to check the highest similarity (> 95%) sequences, but no sequence was observed. Then NFLG reference sequences that covered the currently known HIV-1 group M subtypes were obtained from the Los Alamos National Laboratory (LANL) HIV sequence database (https://www.hiv.lanl.gov). Sequences were subjected to multiple alignments using MAFFT v7.4.8 [15] and trimmed to identical lengths (HXB2: 776–9412 nt). In addition, the Neighbor-Joining phylogenetic tree based on the HIV-1 NFLG sequences involving the currently known HIV-1 subtypes and NFLG sequence of ZJ20202195 was constructed using MEGA program v7.0.2 with 1000 bootstrap replicates under the Kimura 2-parametric model [16] and then were visually edited by FigTree v1.4.1 (http://tree.bio.ed.ac.uk/software/figtree/) and the web-based tool Interactive Tree of Life (iTOL) v6.0 [17]. The NFLG phylogenetic tree revealed that the NFLG of ZJ20202195 had high homology with CRF01_AE reference sequences, forming a branch with high support (bootstrap value of 100). Moreover, ZJ20202195 and CRF01_AE cluster g4a strain (GenBank ID: JX112796) formed an obvious branch distantly related to other CRF01_AE strains, suggesting that ZJ20202195 may be homologous to CRF01_AE cluster g4a strain (Fig. 1A).

**Fig. 1:**
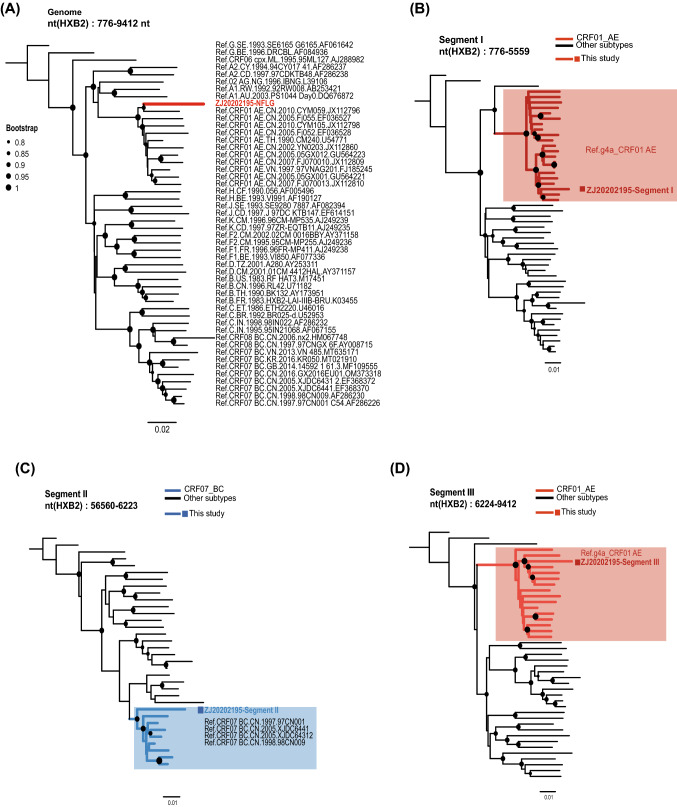
Phylogenetic analysis of the NFLG and recombinant segments of ZJ20202195. **A** The neighbor-joining phylogenetic trees of ZJ20202195 based on the currently known HIV-1 subtype NFLG reference sequences. **B**–**D** The neighbor-joining phylogenetic trees of three recombinant segments of ZJ20202195 based on the main HIV-1 subtype reference sequences and some selected CRF01_AE and CRF07_BC reference sequences. The neighbor-joining trees were reconstructed by MEGA program v7.0.2 with 1000 bootstrap replicates under the Kimura 2-parametric model. Only bootstrap values ≥ 80% were displayed on the corresponding nodes. HIV-1 subtype G (GenBank ID: AF061642) was chosen as an out-group in the rooted tree. The scale bar represents the genetic distance

To determine the recombination breakpoints and structure of the NFLG sequence of ZJ20202195, Similarity Plotting (SimPlot) v3.5.1 [18], Recombination Detection Program (RDP) v4.1.0 [19], and jumping profile Hidden Markov Model (jpHMM) (http://jphmm.gobics.de/submission_hiv) [20] were performed. First, to determine the similarity between the query sequence (ZJ20202195) and different HIV-1 subtype reference sequences, similarity plots and bootscanning analyses were performed using SimPlot v3.5.1 under a sliding window with 400 nts moved in 20-nts steps. The results revealed that ZJ20202195 were more similar to the CRF01_AE reference sequence (GenBank ID: U54771) throughout a genome predominantly, and only a short gene segments located between pol and env genes (about 600 bp), which were more similar to CRF07_BC references sequence (GenBank ID: AF286226) (Fig. 2A). And then bootstrap support analyses of recombination evidence of ZJ20202195 were performed using RDP4; the result was consistent with those through SimPlot analyses and had high support (Fig. 2B). Furthermore, the NFLG sequence of ZJ20202195 was submitted to jpHMM, and the putative recombination structure inferred from the SimPlot and RDP4 analyses was complemented with sequence inspection to define more precisely breakpoint locations to further confirm the above results. To better visualize the recombination map, we submitted these breakpoints to Recombinant HIV-1 Drawing Tool (https://www.hiv.lanl.gov/content/sequence/DRAW_CRF/recom_mapper.html) and redrew the recombination pattern for ZJ20202195 referred to the location of the reference sequence HXB2 (Fig. 2C). These findings amply proved that the NFLG sequence of ZJ20202195 was a recombinant formed CRF01_AE and CRF07_BC subtypes in which the CRF01_AE subtype was the absolute genomic backbone and inserted a small fragment of the CRF07_BC subtype. Two breakpoints delimited three recombinant segments, of which the Segment I (HXB2:776–5559 nt) and Segment III (HXB2:6224–9412 nt) were from the CRF01_AE subtype, located in gag-pol-partial vif overlap and partial vpu-env-nef-partial 3’-LTR, respectively, and Segment II (HXB2:5560–6223 nt) was from CRF07_BC subtype and located in partial vif overlap-vpr-partial vpu.

**Fig. 2:**
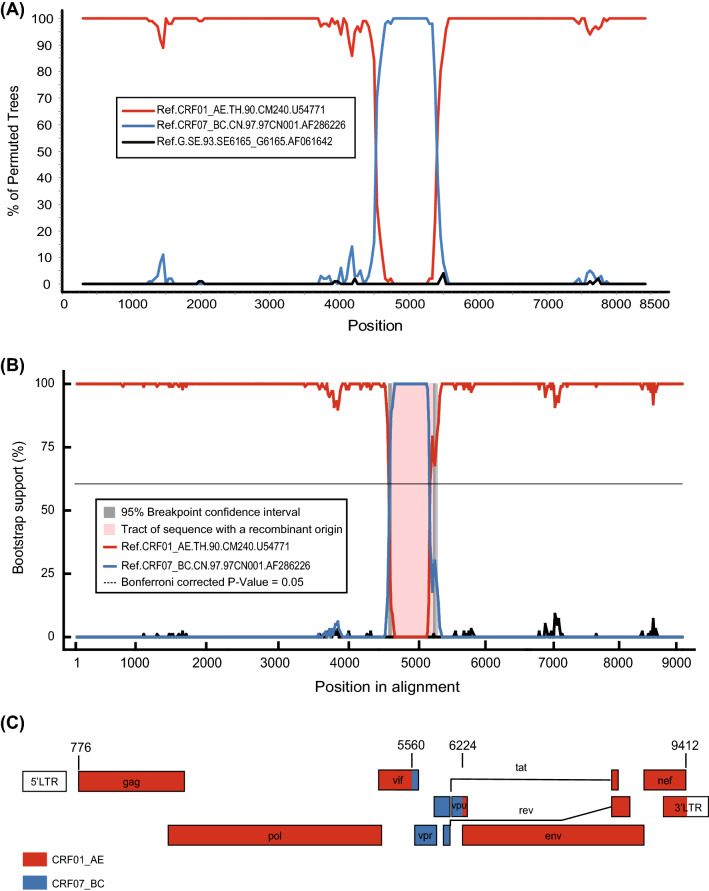
Recombination breakpoints analysis of the NFLG sequence of ZJ20202195. **A** The bootscanning analysis of the NLFG of ZJ20202195. With the NLFG of ZJ20202195 as the query sequence, recombinant breakpoints were scanned by Simplot program v3.5.1. The y-axis represents the percentages of permuted trees, and the x-axis represents each sequence position. **B** The recombination evidence analysis of the NLFG of ZJ20202195 based on RDP4. The y-axis represents the percentages of bootstrap support, and the x-axis represents each sequence position. Other adjustment parameters are shown. **C** Genomic structure map of the NFLG sequence of ZJ20202195 (HXB2:776–9412 nt). The red color represents HIV-1 CRF01_AE subtype, and the blue color represents HIV-1 CRF07_BC subtype in the map. All structural genes and regulatory genes involved in the NFLG sequence of ZJ20202195 were labeled in detail

In addition, phylogenetic trees based on the three recombinant segments were constructed by MEGA program v7.0.2 and were visualized and edited by FigTree and iTOL, indicating that the Segment I and Segment III of the NFLG sequence of ZJ20202195 corresponded well with CRF01_AE cluster g4a strains prevalent in China (bootstrap value = 100%) (Fig. 1B/D**)**, and Segment II formed a general supported (bootstrap value < 90%) monophyletic cluster with CRF07_BC reference sequences (Fig. 1C).

Overall, in this study, we reported a novel HIV-1 unique recombinant form (CRF01_AE/CRF07_BC) in Zhejiang Province, through different recombination and phylogenetic analyses. Zhejiang Province is located in the economically developed Yangtze River Delta region of China, adjacent to Shanghai, Jiangsu, and other provinces or cities, with a frequent population flow. In addition, due to the high variation of HIV-1, the characteristics of different HIV-1 subtypes in Zhejiang Province are diverse due to geographical distributions, transmission patterns, and risk groups, creating a considerable challenge for intervention. Molecular epidemiological surveillance data of Zhejiang Province in 2021 showed that HIV-1 subtypes with more than 15 known and a certain proportion of URFs (accounting for 10%), among which CRF01_AE and CRF07_BC accounted for about 75% (data have not been published), providing favorable conditions for recombination and mutation of HIV-1.

In this study, the recombination pattern of the NFLG sequence of ZJ20202195 was significantly different from the URFs reported until today. The recombination breakpoint of most of these URFs occurred in the structural genes (*gag, pol, and env* gene regions), while the NFLG sequence of ZJ20202195 reported in this study with CRF01_AE subtype as the genomic backbone showed a short fragment inserted from CRF07_BC subtype in the regulatory gene region (vif-vpu) (about 600 bp). Currently, the widely used methods for identification of the HIV-1 subtype are mainly based on partial short structural gene sequences (*gag, pol, env*) [21], causing the misjudgment and limitations of the HIV-1 subtype analysis and limiting the identification of epidemiological characteristics and recombination breakpoints. Therefore, it is necessary to establish a more extensive and practical method for the detection and identification of HIV recombinant strains and provide a scientific basis for the accurate determination and evolution of HIV-1 recombinant strains.

## Sequence data

The nucleotide sequence of ZJ20202195 has been submitted to GenBank with accession no. ON959205.
